# Association between common vaginal and HPV infections and results of cytology test in the Zhoupu District, Shanghai City, China, from 2014 to 2019

**DOI:** 10.1186/s12985-022-01850-x

**Published:** 2022-07-29

**Authors:** Huaping Li, Zhengguang Xiao, Baoling Xing, Suqin Wu, Ying Wang, Zhou Liu, Yanan Zeng, Joseph Cosmas Mushi, Hudie Sun, Ping Li

**Affiliations:** 1 Department of Obstetrics and Gynecology, Shanghai University of Medicine & Health Sciences Affiliated Zhoupu Hospital, No.1500 zhouyuan Road, Pudong New District, Shanghai, 201318 China; 2 Department of Imaging, Tongren Hospital, Shanghai Jiao Tong University School of Medicine, No. 1111 Xianxia Road, Changning District, Shanghai, 200336 China; 3 College of Information and Communication Technologies (CoICT), University of Dar Es Salaam, 14113 Dar es Salaam, Tanzania; 4 College of Medical Instrumentation, Shanghai University of Medicine & Health Sciences, No.279 Zhouzhu, Pudong New District, Shanghai, 201318 China; 5 Sino-European School of Technology, Shanghai University, No.99 Shangda Road, Baoshan District, Shanghai, 200444 China

**Keywords:** Vaginal infection, China, Human papillomavirus, Genotype, ThinPrep cytological test

## Abstract

**Background:**

HPV (human papillomavirus) is an important cause of cervical cancer. Cervical-vaginal infection with pathogens, such as herpes simplex virus (HSV), bacterial vaginosis Trichomonas vaginalis and vaginal candidiasis could be a cofactor. This study aimed to assess the relationship between vaginal infection with HPV genotype and cytology test results and analyze the relationship between vaginal and HPV infections and cervical cancer.

**Methods:**

We performed a district-based study to elucidate the relationship among the vaginal and HPV infections and cervical cancer. We collected the cervical exfoliation data of 23,724 women admitted to the Shanghai Zhoupu Hospital and received ThinPrep cytology test (TCT) and HPV detection between 2014 and 2019.

**Results:**

Total vaginal infection rate was 5.3%, and the HPV-positive group had a slightly higher vaginal infection rate than the HPV-negative group (*P* < 0.01). The incidence rate of cervical intraepithelial neoplasia or cervical cancer with vaginal infection was higher than without vaginal infection (*P* < 0.001).

**Conclusion:**

HPV/vaginal infection-positive women tended to have abnormal results of TCT. Women with vaginal infection were more likely to develop HPV infection. HSV combined with HPV infection was noted as a causal factor for HSIL.

**Supplementary Information:**

The online version contains supplementary material available at 10.1186/s12985-022-01850-x.

## Introduction

Cervical cancer was reported as the fourth most common cancer among women worldwide, ranking after breast cancer, colorectal cancer, and lung cancer [1]. Approximately 0.57 million cases of cervical cancer were diagnosed and 0.31 million cervical cancer-related deaths occurred in 2018 [2]. In China, 98,900 cervical cancer cases were annually diagnosed, accounting for approximately 20% of total new cases globally, ranking seventh and ninth in cancer prevalence and mortality in women, respectively [3]. The persistent infection with carcinogenic human papillomavirus (HPV) is the main cause of triggering the cause of cervical cancer [4]. In China, cytology-based screening combined with HPV-DNA detection is the main diagnostic strategy for cervical cancer [5]. Our previous research indicated that the most prevalent high-risk HPV (HR-HPV) genotypes were HPV52, HPV16, HPV58, HPV53, HPV39, and HPV51 in the Zhoupu District (Shanghai, China) [6]. However, most HPV infections are associated with subclinical or asymptomatic appearance [7]. Why does a small proportion of women infected with the HR-HPV develop clinically significant pre-invasive lesions and cervical cancer? Little is known about the role of vaginal infections in the progression of cervical carcinogenesis and cervical cancer.

Bacterial vaginosis (BV), trichomonas vaginalis (TV), vaginal candidiasis (VC), and herpes simplex virus (HSV) are the common vaginal infections. Recent researches has investigated the vaginal infection as a cofactor in cervical carcinogenesis [5, 8–11]. It has been shown that the risk of the high-grade squamous intraepithelial lesion (HSIL) decreased in TV-positive women, especially in high-risk HPV-positive women [5]. Results of a study showed that vaginal microbiota (VMB) composition was significantly associated with changes in HPV infection status [12]. A previous research demonstrated that an elevated vaginal pH was associated with a 30% greater risk of infection with the low-grade squamous intraepithelial lesion (LSIL) and with multiple HPV types [13]. Given that the VMB composition has been shown to play a role in HPV infection and the rate of HPV clearance, vaginitis may be correlated with the development of cervical cancer secondary to a persistent HPV [14]. Therefore, elucidation of the role of these infectious agents in cervical carcinogenesis has important implications for the management of infected patients and the organization of preventive programs [15]. However, no district-based study has been performed to elucidate the relationship between vaginal and HPV infections and cervical cancer.

The present study aimed to assess the relationship of vaginal infection with HPV genotype and results of cytology test in the Zhoupu District, explore the association between the vaginal infection and HPV genotype, and analyze the relationship between the vaginal and HPV infections and cervical cancer. We retrospectively analyzed the data from a tertiary hospital in Shanghai.

## Methods

### Data collection

We collected the cervical exfoliation data of 23,724 women admitted to Shanghai Zhoupu Hospital (Shanghai, China) and received both ThinPrep cytology test (TCT) and HPV detection between 2014 and 2019.

### Ethics statement

The Ethics Committee approved the study of Shanghai Zhoupu Hospital. All patients have signed the written informed consent forms. For patients younger than 18 years old, their parents have signed the written informed consent forms. Confidentiality was ensured during data collection process, and data were analyzed anonymously.

### Cytology test

During the non-menstrual period, samples of exfoliated cervical cells were collected. Experienced cytologists conducted cytology tests based on cervical fluid. The diagnostic results of the TCT were classified according to the Bethesda system [16]. Liquid-based cytology included negative for intraepithelial lesion or malignancy (NILM), LSIL, HSIL, atypical squamous cells of undetermined significance (ASC-US), atypical squamous cells (ASC-H) that HSIL cannot be excluded, atypical glandular cells (AGC), and adenocarcinoma.

Besides, CIN1 and CIN2 + were corresponded to LSIL and HSIL, respectively. ASC-H, ASC-US, and AGC were associated with highly suspicious precancerous lesions. The ASC-US, LSIL, and HSIL were correlated to abnormal TCT results.

### Vaginal infection

According to the diagnostic criteria of the Bethesda system, there are four vaginal infections in liquid-based cytology: BV, TV, HSV, and VC.

### HPV genotyping

Using HPV typing test kits (PCR + membrane hybridization), HPV genotyping was performed on the collected samples (Certificate No. of China Food and Drug Administration (2014): 3,402,188).

In addition, the PCR membrane hybridization could detect 21 HPV genotypes (15 HR-HPV genotypes: 16, 18, 31, 33, 35, 39, 45, 51, 52, 56, 58, 59, 66, and 68; 6 LR-HPV genotypes: 6, 11, 42, 43, 44, and 81), through reverse dot hybridization and envelope specific probe membrane hybridization.

### Statistical analysis

All HPV and TCT data collected from 2014 to 2019 were combined into an Excel spreadsheet and then analyzed and plotted using the R platform (www.r-project.org) (v3.2.0) and R packages.

The secular trends for TCT and HPV-positive infection rates and their distribution in different age groups were analyzed during 2014–2019 using the Student’s t-test. Comparisons between TCT results and HPV infection subtypes were performed. *P* < 0.001 was considered statistically significant.

## Results

### The overall HPV prevalence and TCT type distribution

**Fig. 1:**
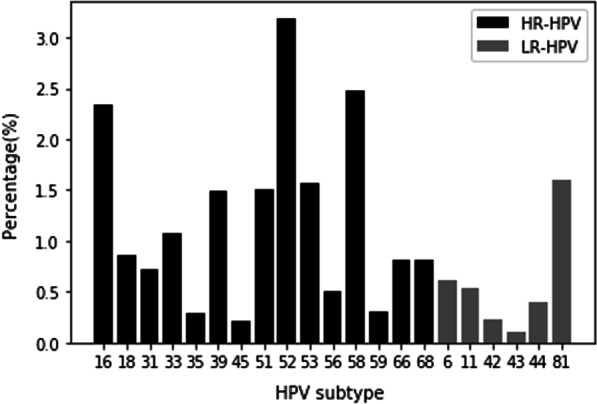
Distribution of HPV genotypes detected in participants. High risk HPV (HR-HPV) in dark color has a higher infection rate than low risk HPV (LR-HPV) in grey color

Overall, HPV-positive infections were detected in 16.08% (3,816/23,724) of patients. In addition, 21 genotypes were identified, including HPV16, HPV18, HPV31, HPV33, HPV35, HPV39, HPV45, HPV51, HPV52, HPV56, HPV58, HPV59, HPV66, HPV68, HPV6, HPV11, HPV42, HPV43, HPV44, and HPV81 (Fig. 1). The most prevalent HR-HPV types were HPV52 (3.19%, 756/23,724), HPV58 (2.47%, 586/23,724), and HPV16 (2.34%, 555/23,724). HPV81 (1.66%, 379/100) was dominant among LR-HPV (LR-HPV) types. The other genotypes had a frequency of ≤ 1.6% (Fig. 1).

Table 1 shows the distribution of four vaginal infections (BV/TV/HSV/VC) separately for HR-HPV and LR-HPV types; at different precancerous stages. According to the five precancerous stages (AGC/ASC/HSIL/LSIL/NILM). Details of each distribution for every BV/TV/HV/CV vaginal infection will be shown in Additional file 1.

**Table 1:** Distribution of four vaginal infections (BV/TV/HSV/VC) at different precancerous stages

HPV subtype	AGC, n (%)	ASC, n (%)	HSIL, n (%)	LSIL, n (%)	NILM, n (%)
HPV16	0(0)	162(29.19)	51(9.19)	23(4.14)	319(57.48)
HPV18	0(0)	56(27.32)	4(1.95)	7(3.41)	138(67.32)
HPV31	0(0)	43(25.15)	4(2.34)	6(3.51)	118(69.01)
HPV33	0(0)	71(27.84)	13(5.1)	10(3.92)	161(63.14)
HPV35	0(0)	18(26.09)	2(2.9)	4(5.8)	45(65.22)
HPV39	1(0.28)	85(24.01)	3(0.85)	13(3.67)	252(71.19)
HPV45	0(0)	14(26.42)	4(7.55)	1(1.89)	34(64.15)
HPV51	1(0.28)	83(23.38)	4(1.13)	11(3.1)	256(72.11)
HPV52	1(0.13)	207(27.38)	16(2.12)	27(3.57)	505(66.8)
HPV53	0(0)	90(24.13)	6(1.61)	18(4.83)	259(69.44)
HPV56	0(0)	35(28.93)	1(0.83)	8(6.61)	77(63.64)
HPV58	1(0.17)	140(23.89)	26(4.44)	21(3.58)	398(67.92)
HPV59	0(0)	17(24.29)	1(1.43)	4(5.71)	48(68.57)
HPV66	0(0)	51(26.15)	2(1.03)	7(3.59)	135(69.23)
HPV68	0(0)	54(28.12)	2(1.04)	5(2.6)	131(68.23)
HPV6	0(0)	50(34.01)	1(0.68)	7(4.76)	89(60.54)
HPV11	0(0)	32(25.4)	0(0)	5(3.97)	89(70.63)
HPV42	0(0)	18(33.33)	1(1.85)	2(3.7)	33(61.11)
HPV43	0(0)	8(30.77)	1(3.85)	1(3.85)	16(61.54)
HPV44	0(0)	27(28.42)	0(0)	1(1.05)	67(70.53)
HPV81	1(0.26)	72(19)	2(0.53)	9(2.37)	295(77.84)

The first 15 types were HR-HPV subtypes and the last 6 types were LR-HPV subtypes

*AGC* atypical glandular cells, *ASC* atypical squamous cells of undetermined significance (ASC-US) and atypical squamous cells that HSIL cannot be excluded (ASC-H), *HSIL* high-grade squamous intraepithelial lesion, *HSIL* low-grade squamous intraepithelial lesion, *NILM* Liquid-based cytology included negative for intraepithelial lesion or malignancy

### The overall status of HPV and vaginal infections

Among 23,724 women, there were 1,265 vaginal infection-positive women and 22,459 vaginal infection-negative women. The total vaginal infection rate was 5.3% (1,265/23,724). The HPV-positive group had a slightly higher vaginal infection rate (6.4% > 5.1%) than the HPV-negative group (*P* < 0.01) (Table 2), indicating that women with HPV infection are more likely to develop a vaginal infection.

**Table 2:** Characteristics of 23,724 women with and without HPV infection

	HPV-positive (n = 3816)	HPV-negative (n = 19,908)	*P* value	OR (95% CIs)
*Age (years old)*				
Mean (SD)	36.7(11.34)	37.08(12.03)		
Median (Min, Max)	34.0(15–87)	36.7(16–94)		
< = 30	1446(37.9%)	7317(36.8%)	**0.0001**	1.0(1.0–1.1)
30–40	952(24.9%)	5719(28.7%)	0.529	0.8(0.8–0.9)
> = 40	1418(37.2%)	6872(34.5%)	0.046	1.1(1.0–1.2)
*Vaginal infections*				
Positive	243(6.4%)	1022(5.1%)	**0.0021**	1.26(1.1–1.5)
Negative	3573(93.6%)	18,886(94.9%)		
*TCT*				
Normal	2609(68.4%)	16,613(83.4%)	**0.0000**	0.43(0.4–0.5)
Abnormal	1207(31.6%)	3295(16.6%)		
*Pathology*				
CIN	235(6.2%)	49(0.2%)	0	
Cervical cancer	0	1(0%)		
Highly-suspected precancerous lesions	972(25.5%)	3245(16.3%)		

**0.0001**: Among women who were younger than 30 years old, 37.9% were HPV-positive and 36.8% were HPV-negative (P < 0.001), **0.0021**: The HPV-positive group had a slightly higher vaginal infection rate (6.4%>5.1%) than the HPV-negative group (P < 0.01), **0.0000**: there was a significant difference in abnormal TCT results between the HPV-positive group and the HPV-negative group (P< 0.001)

*OR (95% CIs)* Odd Ratio (95% Confidence Intervals)

The HPV infection rate in the vaginal infection-positive group (80.8%) was higher than that in the vaginal infection-negative group (15.9%) (*P* < 0.001) (Table 3), demonstrating that women with vaginal infection are also more likely to develop HPV infection. Therefore, it could be speculated that there could be consistency or a synergic interaction between HPV infection and vaginal infection.

**Table 3:** Characteristics of 23,724 women with and without vaginal infection

	Vaginal infection-positive (n = 1265)	Vaginal infection-negative (n = 22,459)	*P* value	OR (95% CIs)
*Age (years old)*				
Mean (SD)	34.4(9.5)	36.9(11.5)		
Median (Min, Max)	32(16–67)	34(15–94)		
< = 30	543(42.9%)	8220(36.6%)	0.0088	0.2(0.2–0.3)
30–40	354(28.0%)	6317(28.1%)	0.5283	0.2(0.2–0.2)
> = 40	368(29.1%)	7922(35.3%)	**0.0000**	0.2(0.1–0.2)
*HPV infections*				
Positive	243(19.2%)	3573(15.9%)	**0.0021**	1.3(1.1–1.5)
Negative	1022(80.8%)	18,886(84.1%)		
*TCT*				
Normal	1222(96.6%)	18,000(80.1%)	**0.0000**	7.0(5.2–9.6)
Abnormal	43(3.4%)	4459(19.9%)		
*Pathology*				
CIN	7(0.6%)	277(1.2%)	0.0259	
Cervical cancer	0	1(0%)		
Highly-suspected precancerous lesions	36(2.8%)	4181(18.6%)		

**0.0000**: 29.1% of women with vaginal infection were older than 40 years, while 35.3% of women without vaginal infection were older than 40 years (P < 0.001), **0.0021**: The HPV infection rate in the vaginal infection-positive group (80.8%) was higher than that in the vaginal infection-negative group (15.9%) (P < 0.001), **0.0000**: The difference was also noted between the vaginal infection-positive group and the vaginal infection-negative group (P < 0.001)

*OR (95% CIs),* Odd Ratio (95% Confidence Intervals)

### The association of HPV/vaginal infections and age

The mean age of the HPV-positive women was 36.7 ± 11.3 (range, 15–87) years old, while the mean age of the HPV-negative women was 37.08 ± 12.03 (range, 16–94) years old (Table 2). The mean age of vaginal infection-positive women was 34.4 ± 9.5 (range, 16–67) years old. The mean age of vaginal infection-negative women was 36.9 ± 11.5 (range, 15–94) years old (Table 3). The mean age of women with both HPV and vaginal infections was 38.5 ± 11.6 (range, 18–69) years old (Table 4).

**Table 4:** Characteristics of 1265 women with both HPV and vaginal infections

	HPV-positive	HPV-negative	*P*-value	OR (95% CIs)
*Age (years old)*				
Mean (SD)	38.5(11.6)	38.6(11.9)		
Median (Min, Max)	36(18–69)	36(16–83)		
< = 30	109(44.9%)	434(42.5%)	0.0516	1.3(1.1–1.6)
30–40	51(21%)	303(29.6%)	0.0624	0.9(0.7–1.2)
> = 40	83(34.2%)	285(27.9%)	0.2378	1.5(1.2–1.9)
*TCT*				
Normal	223(91.8%)	999(97.7%)	**0.0000**	0.3(0.2–0.5)
Abnormal	20(8.2%)	23(2.3%)		
*Pathology*				
CIN	7(2.9%)	0(0%)	**0.0000**	
Cervical cancer	0	0(0%)		
Highly-suspected precancerous lesions	13(5.3%)	22(2.2%)		

**0.0000**: There was a significant difference in abnormal TCT results between the HPV-positive group and the HPV-negative group, **0.0000**: The number of women diagnosed with cervical intraepithelial neoplasia (CIN) and cervical cancer with HPV and vaginal infections was higher than that of women without HPV/vaginal infections (P<0.001)

OR (95% CIs), Odd Ratio (95% Confidence Intervals)

Among women who were younger than 30 years old, 37.9% were HPV-positive, and 36.8% were HPV-negative (*P* < 0.001) (Table 2). Moreover, 29.1% of women with vaginal infection were older than 40 years, while 35.3% of women without vaginal infection were older than 40 years (*P* < 0.001) (Table 3). The results above revealed no significant difference between different ages and HPV/vaginal infections.

### HPV/vaginal infections and histological and cytological examinations

It was revealed that the number of women diagnosed with cervical intraepithelial neoplasia (CIN) and cervical cancer with HPV and vaginal infections was higher than that of women without HPV/vaginal infections (*P* < 0.001) (Table 4), indicating that HPV with vaginal infections may promote CIN and cervical cancer.

Regarding the results of TCT, there was a significant difference in abnormal TCT results between the HPV-positive group and the HPV-negative group (*P* < 0.001) (Tables 2, 4). In the meantime, a difference was also noted between the vaginal infection-positive group and the vaginal infection-negative group (*P* < 0.001) (Table 3). Thus, women with HPV/vaginal infections tend to have abnormal TCT results.

### HPV types and vaginal infection

Table 5 shows the vaginal infection rates for different HPV types. HPV66 (11.28%) was the most prevalent HR-HPV type among single infections, followed by HPV59 (10.0%), HPV42 (9.26%), HPV31 (8.19%), HPV52 (7.67%), and HPV6 (7.48%). Besides, 716 cases were diagnosed with double HPV infections, and 256 cases with multiple HPV infections.

**Table 5:** HPV subtype and vaginal infection

HPV subtype	HPV-positive (n)	Trichomonas infection (n, %)	Candida infection (n, %)	Herpes virus infection (n, %)	Bacterial infection (n, %)	Vaginal infection- positive (n, %)	Vaginal infection- negative (n, %)
HPV66	195	2(1.03)	14(7.18)	0(0)	6(3.08)	**22(11.28)**	173(88.72)
HPV59	70	0(0)	3(4.29)	0(0)	4(5.71)	7(10.0)	63(90)
HPV31	171	1(0.58)	8(4.68)	1(0.58)	4(2.34)	14(8.19)	157(91.81)
HPV52	756	4(0.53)	30(3.97)	1(0.13)	23(3.04)	58(7.67)	698(92.33)
HPV68	192	2(1.04)	6(3.12)	0(0)	6(3.12)	14(7.29)	178(92.71)
HPV35	69	0(0)	2(2.9)	0(0)	3(4.35)	5(7.25)	64(92.75)
HPV16	555	2(0.36)	23(4.14)	1(0.18)	12(2.16)	38(6.85)	517(93.15)
HPV51	355	4(1.13)	8(2.25)	1(0.28)	12(3.38)	23(6.48)	332(93.52)
HPV58	586	4(0.68)	20(3.41)	1(0.17)	13(2.22)	38(6.48)	548(93.52)
HPV45	53	0(0)	2(3.77)	0(0)	1(1.89)	3(5.66)	50(94.34)
HPV39	354	1(0.28)	7(1.98)	1(0.28)	11(3.11)	20(5.65)	334(94.35)
HPV33	255	1(0.39)	7(2.75)	0(0)	6(2.35)	14(5.49)	241(94.51)
HPV18	205	2(0.98)	6(2.93)	0(0)	3(1.46)	11(5.37)	194(94.63)
HPV53	373	2(0.54)	8(2.14)	0(0)	7(1.88)	17(4.56)	356(95.44)
HPV56	121	0(0)	3(2.48)	0(0)	2(1.65)	5(4.13)	116(95.87)
HPV42	54	1(1.85)	2(3.7)	0(0)	2(3.7)	5(9.26)	49(90.74)
HPV6	147	2(1.36)	4(2.72)	0(0)	5(3.4)	11(7.48)	136(92.52)
HPV11	126	1(0.79)	5(3.97)	0(0)	3(2.38)	9(7.14)	117(92.86)
HPV81	379	0(0)	12(3.17)	1(0.26)	10(2.64)	23(6.07)	356(93.93)
HPV44	95	0(0)	2(2.11)	0(0)	2(2.11)	4(4.21)	91(95.79)
HPV43	26	0(0)	0(0)	0(0)	0(0)	0(0)	26(100)
Single infection	2844	13(0.46)	86(3.02)	2(0.07)	74(2.6)	175(6.15)	2669(93.85)
Double infections	716	5(0.7)	28(3.91)	1(0.14)	17(2.37)	51(7.12)	665(92.88)
Triple infections	186	0(0)	7(3.76)	1(0.54)	6(3.23)	14(7.53)	172(92.47)
Quadruple infections	53	0(0)	1(1.89)	0(0)	1(1.89)	2(3.77)	51(96.23)
Multiple infections	17	1(5.88)	1(5.88)	0	1(5.88)	3(17.65)	14(82.35)

**22(11.28)**: HPV66 was the most prevalent HR-HPV type among single infections

The first 15 types were HR-HPV subtypes and the last 6 types were LR-HPV subtypes

No vaginal infection was found in none of the cases with HPV43-positive infection. Although most women were single-type HPV-positive infections (74.53%, 2,844 of 3,816), there was almost the same percentage of vaginal-positive infection (6.15%) in double-type (7.12%) and triple-type (7.53%) HPV-positive infections. Moreover, more vaginal infections (17.65%) were found in the multiple-type HPV-positive infections.

**Fig. 2:**
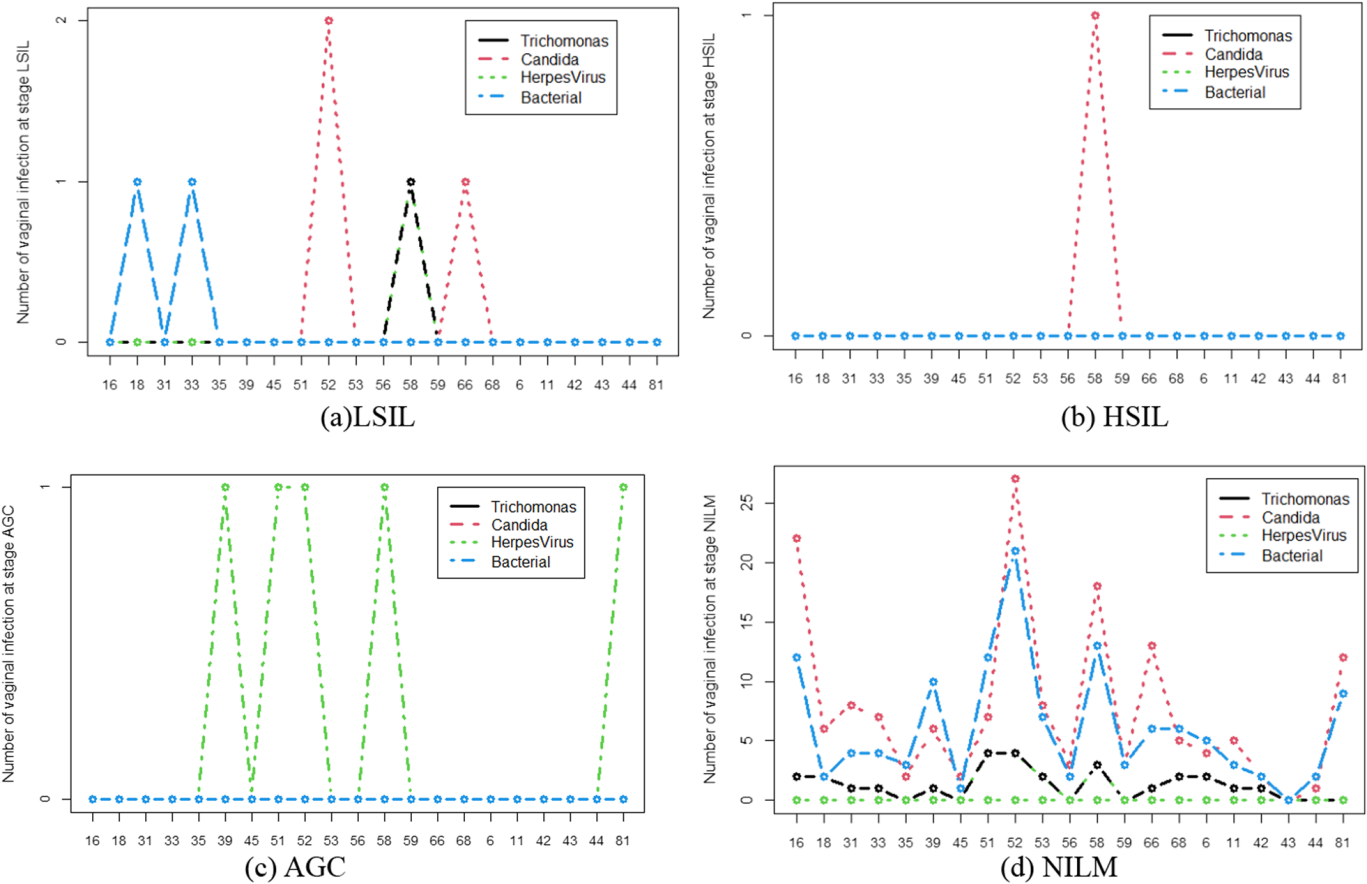
Distribution of HPV types in vaginal infection. **a** Distribution of HPV types with vaginal infection-positive in LSIL. **b**. Distribution of HPV types with vaginal infection-positive in HSIL. **c**. Distribution of HPV types with vaginal infection-positive in AGC. **d**. Distribution of HPV types with vaginal infection-positive in NILM

Figure 2 illustrates the distribution of all HPV subtypes for different types of vaginal infections at four precancerous stages. Figure 2a shows the distribution when women were diagnosed with LSIL. HPV18 and 33 were both found in the BV infections, HPV58 was found in both TV and HSV infections, and HPV52 and 66 were both found in the Candida infections. Women with HSIL were only infected by HPV58 and Candida (Fig. 2b). Women with AGC were infected by HSV and HPV39, 51, 52,81(Fig. 2c). As shown in Fig. 2d, in NILM women with HPV43 were not found with any infections. Except for HSV, three other vaginal infections were found in the most normal NILM women. Bacteria and Candida infected most NILM women, and Candida infected 27 women with HPV-52.

No vaginal infections were detected in women with ASC and cervical cancer. Details of each distribution for four precancerous stages (LSIL/HSIL/ASC/NILM) will be shown in Additional file 2.

## Discussion

Although most HPV infections are cleared within two years [17], differences in immune responses to HPV and other exogenous factors may increase the risk for HPV infection, persistence, and rapid progression to cervical cancer [18]. Previous research demonstrated that having a vaginal infection complicated with HR-HPV affects the development of CIN in subjects with ASC-US cytology [19]. Vaginal infections are consistently associated with the development of cervical cancer [20]. However, the association between different types of vaginal and HPV infections and cervical precancerous lesions has remained elusive. In the present study, we performed a district-based study to elucidate the relationship between the different types of vaginal and HPV infections and cervical cancer. The mean age of HPV-positive women was 36.7 ± 11.3 years old, the mean age of women with HPV and vaginal infections was 38.5 ± 11.6 years old, and the results showed no significant difference between different ages and HPV/vaginal infections. Moreover, the association between HPV infection, abnormal cervical cytology, and different vaginal infections have been considered. The most prevalent types of HR-HPV were HPV52 (3.19%, 756/23, 724), HPV58 (2.47%, 586/23, 724), and HPV16 (2.34%, 555/23, 724), and HPV81 (1.66%, 379/100) was dominant among LR-HPV types. Among 23,724 women, the total vaginal infection rate was 5.3% (1,265/23,724). The HPV-positive group had a slightly higher vaginal infection rate than the HPV-negative group (*P* = 0); the HPV infection rate in the vaginal infection-positive group (80.8%) was higher than that of the vaginal infection-negative group (15.9%) (*P* = 0). The results revealed that women with vaginal infection were more likely to develop HPV infection. The risk of CIN or cervical cancer with vaginal infection was higher than without vaginal infection (*P* < 0.001). The results suggested that women with both HPV and vaginal infections tended to have abnormal TCT results. When women were diagnosed with LSIL, HPV16 and HPV33 were prevalent in bacterial infections, HPV58 was prevalent in the TV, and HPV52 and HPV66 were prevalent in the VC. Women with HSIL were all infected, and they were infected by the HPV39, HPV51, HPV52, HPV81, and the HSV. Women with AGC were all infected by HPV58 and Candida. Overall, the data supported the role of HSV infection as a causal factor for HSIL, and the results also showed that interaction between HPV infection and vaginal infection might explain a substantial proportion of the high risk of infection found in the study.

Only a small proportion of women with HPV infection can persist and progress to cervical cancer [21], in which cofactors may act in combination with HPV, and the combined effect of HSV and HPV has been reported as a cofactor for cervical cancer [22]. Our study revealed that the women with HSIL were all infected, and they were infected by the HPV39, HPV51, HPV52, HPV81, and the HSV, suggesting that interaction between HPV and HSV infections may affect the development of CIN. A significantly higher positivity of HSV2 has been found in women with cervical dysplasia and carcinoma-in-situ [23] compared with HPV DNA-positive normal controls, and the seropositivity of HSV2 has increased the risk of development to squamous cell carcinoma or adenocarcinoma [22]. In India, HSV diagnosed by cytological smear was mainly correlated to CIN and cervical cancer, and had a higher affinity than HPV [24]. Immunocytochemistry suggested that HSV2 could be associated with squamous cell carcinoma cervix and carcinoma in situ [25], demonstrating that HSV2 could act in conjunction with HPV infection in cervical malignant transformation [26]. HSV genes may be necessary for the initiation, but not progression in cervical malignant transformation as proposed by the "hit and run" mechanism [27], and induced the accumulation of genetic abnormalities and destroyed the stability of host genome, indicating the role of HSV in the malignant transformation [28, 29]. However, some studies have shown the lack of correlation between HSV-1 or HSV-2 and the occurrence of cervical cancer [30] [31]. Our study found that women with HSIL were all infected by HPV39, HPV51, HPV52, and the HSV, in which HSV may as a cofactor with the HR-HPV to promote cervical malignant transformation.

To our knowledge, BV is the most common type of vaginal infection in adult women. It is characterized by the overgrowth of anaerobic bacteria and elevated vaginal pH (> 4.5) [32]. The bacterial infection reportedly cofactors in cervical carcinogenesis [33]. There was a correlation between BV and the relapse of CIN2 + lesions after LEEP [34]. In our study, among women diagnosed with LSIL, the HR-HPV16 and HR-HPV33 were dominant in bacterial infections, while women with bacterial infections were all infected by the LR-HPV 44 and LR-HPV81, and in NILM women were diagnosed with HR-HPV31, HR-HPV33, and HR-HPV59. There was no bacterial infection in women with AGC and HSIL. BV may be acted as a cofactor with HPV in the LSIL, which may affect cervical carcinogenesis. BV may be associated with high levels of anaerobic organisms, which can damage the vaginal epithelium and increase the risk of HPV infection [35]. However, it was noted that HPV-positive women with BV had a limited association with HSIL and AGC. Previous study, different from ours, reported that Gardnerella continuously emerged as a risk factor for CIN2 + development and progression [36]. The association may be tied to the ability of Gardnerella to be immunosuppressive in the cervicovaginal region [37].

TV and VC are common vaginal infections. Among women diagnosed with LSIL, women with TV were all infected by the HR-HPV58, those with Candida were all infected by HR-HPV52 and HR-HPV66, women with AGC were all infected by HPV58 and Candida, and there were no NILM women infected by TV and HPV43. Our study showed TV and 273 VC were associated with HR-HPV infection, consistent with other studies [15, 38]. Trichomoniasis can damage the vaginal epithelium, degrade cervical mucus, and cleave immunoglobulin A [39]. Some strains of Trichomonas vaginalis carry their viruses that amplify inflammatory responses [40]. A study showed that Trichomonas vaginalis infection increased HPV infection by 6.5 times, increasing the risk of CN [41]. Candida can enhance proteolytic activity and antigen modulation, enabling micro-organisms to penetrate the mucosal surface and induce mucosal swelling, erythema, and exfoliation of cells [42]. However, HPV-positive women with co-existing TV and VC infections in our study were not associated with HSIL. The findings of our study suggested that TV and VC infections were likely to play some causal roles in CIN, while they were unlikely to have an association with HSIL. A study showed that co-infection with VC and/or TV did not enhance the carcinogenic effects of HPV on the cervix, and VC and TV could be secondary infections of the malignant growth rather than any causal roles [43]. Meanwhile, BV, TV, and VC were not associated with 6-month persistent non-HPV16/18 infections (odds ratio (OR):1.02, 95% confidence interval (CI): 0.62–1.69) [44].

Since the use of many culture and staining techniques to improve the laboratory diagnosis reliability of BV, Candida spp., and TV. The highest percentage of positives in TV was found by examination of Papanicolaou-stained cervical smears, compared with microscopy of Giemsa (GS) applied in 1958 and acridine-orange (AO) stained smear in 1958 [45]. Papanicolaou (Pap) smears analysis based on the Bethesda system was recommended as a BV screening method for adolescents and women in 2014 [46, 47].With the progress of laboratory testing technology for vaginitis, more detection techniques are applied in clinical laboratories. The Centers for Disease Control and Prevention recommends the prevention of molecular tests [48]. The diagnosis of vaginitis is usually used by clinical manifestations, wet mount, Amsel criteria or laboratory tests [48–50]. However, Amsel's criteria are highly subjective and open to interpretation [51].Vaginal swab Gram stain with estimates of numbers of microbial flora is an alternative method for diagnosing BV [52].The BD Affirm VPIII microbial identification test system was widely used by many clinical laboratories for gynecologists to diagnose vaginal infections [53]. A study showed that the diagnostic accuracy of the combined nucleic acid amplification test (NAAT) construct was approximately 20 to 25% higher than that of the Affirm VPIII,when modeled in populations with various prevalences of infectious vaginitis [54]. Several studies demonstrated that using a combination of NAA tests for BV, Candida spp., and TV can result in a significant increase in the accuracy of diagnosis of women presenting with vaginitis syndrome [54, 55]. The GenProbe Aptima Trichomonas vaginalis assay (ATV) was the Food and Drug Administration (FDA) approved NAAT for TV, data form FDA clearance established the ATV’s sensitivity was 98.9% for TV [56]. Although this new detection method performs well, a more comprehensive understanding of its real performance is limited by the known limitations of the reference method [57].

However, there were some limitations in our study. First, this study was carried out in only a single center in the Zhoupu District, and additional multicenter data need to be collected. Second, although HPV genotypes, infection types, and cytological test results are biologically independent, their possible correlation should be further detected. Third, The detecting method of cervical-vaginal infection with HSV, BV, TV and VC has limitations. We plan to use the machine learning method to examine the relationship between HPV subtypes and pathological types, including cervicitis, cervical precancerous lesions, CIN2/3, and cervical cancer.

## Conclusions

The HPV/vaginal infection-positive women had abnormal TCT results. Women with vaginal infection were more likely to develop HPV infection. HSV infection was noted as one of the causal factors for HSIL. The observed associations among common vaginal and HPV infections and the cytology test results are worthy of further investigation. Elucidation of the role of these infectious agents in cervical carcinogenesis has important implications for the management of infected patients and the organization of preventive programs, highlighting the significance of the results of the current study. Further district-based research on cervical carcinogenesis is required to elucidate the etiology of such multifactorial diseases and to more reliably diagnose and treat cervical cancer that causes high morbidity and mortality among women globally.

## Supplementary Information

**Additional file 1.** Supplement 1: Distributions of four vaginal infections for 21 HPV subtypes at five precancerous stages.

**Additional file 2.** Supplement 2: Distributions of five precancerous stages for 21 HPV subtypes at four vaginal infections.

## Data Availability

The data were collected from Zhoupu District Hospital in Shanghai City. We are grateful to their generous help. The data can be freely shared. The materials were purchased from Hybribio Biotechnology Co., Ltd. (China).

## Abbreviations

HPV: Human papillomavirus
HSV: Herpes simplex virus
BV: Bacterial vaginosis
TV: Trichomonas vaginalis
VC: Vaginal candidiasis
TCT: ThinPrep cytology test
AGC: Atypical glandular cells
LSIL: Low-grade Squamous Intraepithelial Lesion
HSIL: High-squamous intraepithelial lesion
VMB: Vaginal microbiota
HR-HPV: High-risk HPV
LR-HPV: Low-risk HPV
NILM: Negative for intraepithelial lesion or malignancy
ASC-US: Atypical squamous cells of undetermined significance
ASC-H: Atypical squamous cells that HSIL cannot be excluded
CIN: Cervical intraepithelial neoplasia
