# Correction: Association between common vaginal and HPV infections and results of cytology test in the Zhoupu District, Shanghai City, China, from 2014 to 2019

**DOI:** 10.1186/s12985-022-01874-3

**Published:** 2022-09-06

**Authors:** Huaping Li, Zhengguang Xiao, Baoling Xing, Suqin Wu, Ying Wang, Zhou Liu, Yanan Zeng, Joseph Cosmas Mushi, Hudie Sun, Ping Li

**Affiliations:** 1 Department of Obstetrics and Gynecology, Shanghai University of Medicine and Health Sciences Affiliated Zhoupu Hospital, No. 1500 Zhouyuan Road, Pudong New District, Shanghai, 201318 China; 2 Department of Imaging, Tongren Hospital, Shanghai Jiao Tong University School of Medicine, No. 1111 Xianxia Road, Changning District, Shanghai, 200336 China; 3 College of Medical Instrumentation, Shanghai University of Medicine and Health Sciences, No. 279 Zhouzhu, Pudong New District, Shanghai, 201318 China; 4 College of Information and Communication Technologies (CoICT), University of Dar Es Salaam, 14113 Dar es Salaam, Tanzania; 5 Sino-European School of Technology, Shanghai University, No. 99 Shangda Road, Baoshan District, Shanghai, 200444 China

## Correction to: Virology Journal (2022) 19:127 10.1186/s12985-022-01850-x

Following publication of the original article [1], we have been informed that the order of the 3rd and 4th affiliation was incorrectly listed.

The order of the affiliations has been updated above and the original article [1] has been corrected.

## References

[1] Li H (2022). Association between common vaginal and HPV infections and results of cytology test in the Zhoupu District, Shanghai City, China, from 2014 to 2019. Virol J.

